# Materials for Filling Teeth

**Published:** 1878-10

**Authors:** I. P. Wilson

**Affiliations:** Burlington


### THE DENTAL REGISTER.

VOL. XXXII.] OCTOBER, 1878. No 10.

### MATERIALS FOR FILLING TEETH.

BY DR. I. P. WILSON, BURLINGTON.

*Read before the Iowa State Dental Society.*

It is unfortunate that we do not as a profession agree upon the best material for filling teeth. True, a majority of prominent dentists are united in recommending that chemically pure gold is the best filling material, while a few distinguished operators tell us, that amalgam, Hill's stopping, and the so-called oxy-chlorides of zinc are for various reasons more satisfactory, and more reliable as filling materials. This difference of opinion is causing warm discussions, and unkind words are sometimes used in condemning men for what they honestly believe. This is certainly wrong, besides being the weakest kind of argument. It even savors of a suspicion that the uncharitable one is in the wrong. It is better, therefore, not to use hard language, as hard argument will answer a better purpose. But I fear discussion of the subject between men of high professional attainments has resulted in evil, because students of dentistry, and practitioners of limited experience, and may I not say, too, of limited ability, are confused and sometimes led astray by the conflicting theories of these men who are regarded as worthy examples. Honest men who want to do right, and yet are lacking in experience or ability, find that they succeed quite well with amalgam, but with gold they have failure after failure. They read the writings of men who are high up in the profession, and find that they, likewise, succeed with amalgam, but with gold they have miserable failures. What is the result? Instead of our young men aspiring to the high position of artists in the profession, amalgam with many of them is the height of their ambition. They have been unable to reconcile the inharmonious theories of these eminent men, and so they are left to act as they please, and usually they please to act the easier way, knowing at the same time that there is high authority for the course of action upon which they decide. If luckily they aspire to proficiency as fine operators with gold, there are many distinguished witnesses to prove the wisdom of their course. And so let them adopt either method of practice, and there are professors in our Dental Colleges to sanction their practice, and call it sound. This is a license that ought not be given young men. It is, therefore, unfortunate, as I have said, that our profession is not a unit on this important question. While plastic fillings have a place and an important place, too, in our profession, yet gold, ever precious, ever pure, ever free from corrosion is the crowning material of all for filling teeth. But why should such widely different results be reported by different operators? Is it in their different modes of operating or is it in the material that is used? These are questions worthy of our consideration, and questions, I may say, that I feel a hesitation in asking, yet the truth is what we want, and that is sometimes difficult to obtain. It is impossible for us to reach correct conclusions from what our patients tell us. In making this statement, I do not charge them with dishonesty, but with forgetfulness. How often do they tell us that we filled such and such teeth—that the fillings are now out, etc., when our unerring ledger, with its stubborn record, proves that they are mistaken. Is it safe, then, to make a record of the failures of other men, from what our patients have told us? A distinguished advocate of plastic fillings has said that he does not base his conclusions upon the result of his own practice alone, but upon the failures of others. Such a record is unreliable and unjust. We have no right to place an estimate upon the operations of a brother dentist, without we know exactly what we are doing, and how can we know without the record.

When a man tells me that he saves more teeth with amalgam than he does with gold, I am compelled to believe one of two things. Either that he uses amalgam almost exclusively or that he is not skillful as an operator with gold, which is to say that gold in the hands of an inefficient dentist is a poorer material for saving teeth than amalgam. I have only to look back to the first years of my own practice to be convinced that I could have saved more teeth then with amalgam than I did with gold, but had I received the benefits of clinical instruction in a dental college before commencing to practice, as I should have done, I need not here make this acknowledgment. Filling teeth with amalgam reqnires less experience, less manual labor and far less skill than does filling the same teeth with gold. It follows, then, that the unaspiring, the inexperienced, the unskilful, and especially the lazy man is not a friend to gold, and gladly does he bolster himself up with the backing he receives from the few distinguished ones who discard gold and advocate in its place plastic fillings. The question of gold and amalgam as filling materials, is, in my opinion, more a sanitary question than it is a question of saving the teeth. That both of these materials will save teeth, and often save them for a life time is well known by us all. I am inclined to think, as I have said, that we should take a sanitary view of the matter, and looking at the subject from that standpoint, who of us will say that amalgam, subject as it is to corrosion, is as healthful as gold? I have seen lead fillings of many years standing, the teeth in a perfect state of preservation, but the lead was corroded and the teeth were dark as night. All of us have seen teeth that have been preserved for years from salivary calculus. Large cavities are sometimes filled with deposits of this kind, and decay is completely arrested, and yet who of us will argue for a moment that tartar is a healthful material for filling the teeth? Are gold fillings, under any circumstances, ever harmful to the teeth or to the human system? I answer, No. Are amalgam fillings ever injurious to the health? I answer unhesitatingly, Yes. Who of you can call to mind an instance where the largest gold fillings were offensive from corrosion? True, there may be collections of decomposed food upon the fillings and teeth of neglectful persons, but no action will be found upon the precious metal itself. Not usually so with large amalgam fillings in such mouths, except it be on the masticating surfaces of the teeth. Have you ever thought of the amount of amalgam that can be introduced into one set of teeth—say twelve molars? By actual weight I have found that not less than twenty grains are not unfrequently used for a single filling, while twenty-five to thirty grains are sometimes required. Just think of twelve or fifteen dwts. of amalgam in one set of molars, and all this mass in a state of corrosion. No wonder we sometimes find in these cases symptoms of ptyalism, no wonder such teeth present an unsightly appearance. Strange if two hundred grains of amalgam in a corrosive condition is not injurious to health. But I do not take the ultra position of some that amalgam should never be used and that our profession and our patients would be better off if there was no such filling material in use. On the other hand, I believe we would sustain a great loss were we to entirely abandon its use. It has a place, and a large place too, in our profession. I will not preach one thing and practice another. I use amalgam, but I endeavor to use it with proper discretion. If our patients are poor and can not afford gold, it is better, far better, that amalgam or some of the cheaper filling materials be used than to allow the teeth to remain unfilled, and finally be lost. But in such cases I would not "plaster up" a whole row of them, where the cavities are all large. Let the forceps be used on the worst of them, so as to "thin them out," and interrupt the crowded arch—then advise special care in cleansing the teeth and little if any harm may follow. The amount of amalgam it will take to fill the shell of a large molar, will be sufficient to fill a small cavity in every tooth in the mouth. Now I do not think that one large filling, or thirty-two small ones will do any particular harm—on the other hand there may be a vast deal of good accomplished in saving a fragile tooth with amalgam that is needed for mastication, and yet is too fragile to admit of a good gold filling. The same large filling divided into small ones may save a whole set of teeth from ruin. I think that you will see from the foregoing that it is large quantities of amalgam in a crowded condition that I so much depreciate. But there is another important question to be taken into consideration. Amalgam is easily manipulated. Difficult gold fillings weary the operator both physically and mentally. If any one believes amalgam equal to gold, (and the class of men alluded to above are glad to believe so) that material will be used. Here then is a fruitful opening for the character. A valuable material is brought into discredit by its indiscriminate use in the hands of a quack, and because of this some of our most eminent dentists have abandoned its use, while others condemn it and yet use it "on the sly." Better practice what we preach. So I come to you to-day, desiring to give amalgam all the credit to which it is entitled.

When amalgam should be used and when it should not, is a question for the conscientious dentist to decide. There are several things to be taken into consideration—the condition of the teeth to be filled and the financial ability of your patient—are the most important considerations. If a tooth is exceedingly frail, and liable to break away while filling, or subsequently from mastication; I ask, gentleman, are you justifiable in subjecting your patients to two, three, or even four long hours of torture—with rubber dam or tongue-holder applied, and you tugging away at the corner of your patient's mouth—while his numb and aching cheek is firmly grasped, and held by your strong hand, and at the same time your assistant standing by with mallet and muscle to give the poor fellow his full measure of misery? I say are you justifiable in doing this without good assurance of a successful operation? Is saving a tooth for one, two or three years a suitable compensation for the time, suffering and money necessary for its accomplishment with gold, when a plastic filling would have answered as good a purpose—cost much less time, suffering and money? Answered as good a purpose did I say? I will say more, I will charge the operator with robbing his patient of time—of inflicting on him unnecessary pain, and of cause him to incur an undue expense without receiving its value in return, and all for the s[illegible]ke of "riding a hobby." I have reference now to teeth too frail to admit of the necessary pressure for perfect gold fillings, and yet not too frail to be filled successfully with amalgam. I know that some of our best operators claim that any tooth that can be filled at all, can be filled successfully with gold. As a rule, I believe this to be true, yet there are exceptional cases. An egg shell may be filled perfectly with amalgam, Guillois sement, and other kindred pasts, but we would fail were we to attempt the same operation with gold. The one is possible the other impossible.

But in using amalgam the best should be employed. I was taught to believe that to use amalgam at all was almost a crime, and I well remember the first time I employed its use. My patient was poor and could not afford gold, so the posterior teeth were filled with tin foil, except one second superior molar on its buccal surface. The nerve had been removed, and when the time for filling came, I found myself unable to gain access to the cavity, so as to fill it successfully with tin, so after two or three attempts, and as many failures, I despairingly resorted to Lawrence's amalgam. I dismissed my patient feeling satisfied with all my fillings but this one, which for the sake of my reputation I hoped might not come under the notice of any other dentist. Years past, and my patient again made her appearance. I was anxious to know the result, and on examination found the detested amalgam filling the only one in good condition. The tin fillings were all on masticating surfaces, and were worn out. The tooth filled with amalgam had retained its color, (the cavity having been lined with tin foil) and was in a perfect state of preservation. I was most agreeably surprised, and commenced the use of amalgam from that time. A brother dentist recommended Townsend's, so I procured a package of that, which was by no means satisfactory. It is far inferior to any I have ever used. If there was nothing better in the market, the sooner all of us abandoned amalgam entirely the better. I do not wonder at the recantation of the lamented Townsend after giving his amalgam a thorough trial. Tin foil has almost gone into disuse, not because of injurious effects it has upon the teeth or the health, but because it lacks durability on masticating surfaces, and because, too, it requires about as much time and skill as gold, and as skill, as well as time, is money, dentists can not afford to introduce tin for much less compensation than gold, and that being the case gold is chosen.

It is unfair to judge of the merits or demerits of any filling material fiom the results of a few isolated cases. Who of us have not seen not only gold, but amalgam and tin foil fillings of more than a quarter of century standing, and yet in a perfect state of preservation, and who of us have not had our best efforts thwarted with each and all of these materials? The fact is the most skillful operators all have failures. We We are not justified in telling our patients that this or that filling will be a permanent one. We do not know what a year will bring forth. Negligence on the part of the patient —improper food—abuse of the teeth, and various circumstances beyond our control, may conspire to lay in ruins the most skillful work of our hands. Who then should be responsible? Why charge such failures upon the material used? Why ask a dentist to insure his fillings? The thought is preposterous. A few months since a gentleman called at our office to have a tooth filled. Eleven years before I had filled ten molars for him with gold. All without an exception, were in good condition. I was pleased with the result, and calling to my partner, said, "see here, Denise, are ten gold fillings of mine of eleven years standing." My associate took a mouth glass and examined each filling carefully then said as he walked away, "Pretty good teeth to hold those fillings so long." This left-handed compliment was unexpected, and I was a little disappointed, but after a moment's reflection I realized the truth of what he had said, viz.: that success in filling teeth is dependent upon good tooth structure as well as good fillings. There is only one way for us to arrive at correct conclusions with reference to the merits of different filling materials. All such operations should be recorded. Any peculiar condition of the teeth at the time of filling should be accurately noted, and then if you fail you know under what conditions you have failed. I speak of this because we can not safely judge of these things from what our patients tell us. Memory is treacherous, and especially so with reference to this subject. Patients will often tell us of Dr. A.'s failures, or it may be that he will bring the matter nearer home and say that you filled his teeth only a year before and now the fillings are all out; that he came to see what you were going to do about it, etc. Such remarks made in the presence of other patients who are waiting for the first time for your professional services, is exceedingly annoying. Under such circumstances you are very fortunate if you have your recording ledger to refer to, which will settle the correctness of the statement with unerring certainty, not favoring patient nor dentist. Frequently we will find that we have filled some of the teeth before, but the new cavities are found on the opposite side from the old fillings. In such cases our recording ledger is our only protection. But it does not protect a fellow practitioner, and so we can not safely make out a record of facts based upon the testimony of some interested witnesses, and we may greatly wrong a brother dentist by placing an estimate upon his professional skill from such evidenee. I do not in this wish to charge dishonesty upon our patients, for we find honest, conscientious patients every day who are mistaken about this matter. I fear that too many of the reports we read in our dental journals are made up from unreliable evidence of this kind. Many of the glass-tube tests for fillings are likewise unreliable and should not be accepted as positive evidence.

One will build up a convincing theory that gold is the only material healthful for the mouth, another that plastic materials are the only kind compatible with tooth-bone, another that gold and amalgam should never be used in the same mouth, that a galvanic current is created by the two metals, the acids of the mouth being the propelling power, and so we have advocates for every theory, with perhaps some truth in all, but he who mounts upon any theory and rides it as a hobby is fanatical and not wise.

Let us briefly recapitulate. Gold is indispensible as a filling material, free from corrosion, perfect in its adaptation to the walls of the teeth, irresistible to the acids of the mouth and durable as the everlasting hills. Amalgam is valuable for filling large and inaccessible cavities in frail teeth and for filling any cavities in the posterior teeth, which would otherwise go unfilled because of the expense of gold. Other plastic fillings are of great value in sensitive teeth, over exposed pulps and sometimes as so-called permanent fillings. With these considerations before us, what then is the conclusion of the whole matter.—Gold is king.

